# Concept of natural genome reconstruction. Part 4. Integration of extracellular double-stranded DNA fragments into the genome of hematopoietic stem cells and the formation of extrachromosomal intermediates

**DOI:** 10.18699/vjgb-26-18

**Published:** 2026-04

**Authors:** S.G. Oshikhmina, V.S. Ruzanova, G.S. Ritter, E.V. Dolgova, S.S. Kirikovich, E.V. Levites, Y.R. Efremov, T.V. Karamysheva, A.S. Molodtseva, Y.V. Raitsina, O.S. Taranov, S.V. Sidorov, S.D. Nikonov, O.Y. Leplina, A.A. Ostanin, E.R. Chernykh, N.A. Kolchanov, A.S. Proskurina, S.S. Bogachev

**Affiliations:** Institute of Cytology and Genetics of the Siberian Branch of the Russian Academy of Sciences, Novosibirsk, Russia Novosibirsk State University, Novosibirsk, Russia; Institute of Cytology and Genetics of the Siberian Branch of the Russian Academy of Sciences, Novosibirsk, Russia; Institute of Cytology and Genetics of the Siberian Branch of the Russian Academy of Sciences, Novosibirsk, Russia; Institute of Cytology and Genetics of the Siberian Branch of the Russian Academy of Sciences, Novosibirsk, Russia; Institute of Cytology and Genetics of the Siberian Branch of the Russian Academy of Sciences, Novosibirsk, Russia; Institute of Cytology and Genetics of the Siberian Branch of the Russian Academy of Sciences, Novosibirsk, Russia; Institute of Cytology and Genetics of the Siberian Branch of the Russian Academy of Sciences, Novosibirsk, Russia; Institute of Cytology and Genetics of the Siberian Branch of the Russian Academy of Sciences, Novosibirsk, Russia; Institute of Molecular and Cellular Biology of the Siberian Branch of the Russian Academy of Sciences, Novosibirsk, Russia; Institute of Cytology and Genetics of the Siberian Branch of the Russian Academy of Sciences, Novosibirsk, Russia Novosibirsk State University, Novosibirsk, Russia; State Scientific Center of Virology and Biotechnology “Vector” of Rospotrebnadzor, Koltsovo, Novosibirsk region, Russia; Novosibirsk State University, Novosibirsk, Russia City Clinical Hospital No. 1, Novosibirsk, Russia; Novosibirsk Tuberculosis Research Institute, Novosibirsk, Russia; Research Institute of Fundamental and Clinical Immunology, Novosibirsk, Russia; Research Institute of Fundamental and Clinical Immunology, Novosibirsk, Russia; Research Institute of Fundamental and Clinical Immunology, Novosibirsk, Russia; Institute of Cytology and Genetics of the Siberian Branch of the Russian Academy of Sciences, Novosibirsk, Russia; Institute of Cytology and Genetics of the Siberian Branch of the Russian Academy of Sciences, Novosibirsk, Russia; Institute of Cytology and Genetics of the Siberian Branch of the Russian Academy of Sciences, Novosibirsk, Russia

**Keywords:** FISH, whole genome sequencing, integration into the genome, extrachromosomal ring structures, FISH, полногеномное секвенирование, интеграция в геном, экстрахромосомальные кольцевые структуры

## Abstract

To assess the possibility of integrating extracellular double-stranded DNA fragments into the recipient genome of hematopoietic stem cells, a complex substrate was constructed consisting of the entire M13F-AluI-M13R fragment and its two restrictive derivatives, appearing after hydrolysis with restriction endonucleases EcoRI and HindIII: M13F-AluI-EcoRI and M13R-AluI-HindIII. The substrate contained a pBlueScript+ plasmid polylinker sequence, absent in the human genome, which framed the human AluI fragment cloned at the EcoRV site. Human bone marrow cells were treated with the DNA of the constructed complex substrate; taking into account the repair time of pangenomic single-strand breaks, preparations of metaphase plates were obtained. FISH revealed specific fluorescent signals. Simultaneously, DNA isolated from colonies obtained from bone marrow cells treated with a complex substrate was sequenced. Two rounds of sequencing were carried out: whole-genome and selective after targeted hybridization on metal beads. The results obtained indicate that homologous exchange between extrachromosomal and chromosomal DNA is possible. Integration into the genome via the single-strand annealing mechanism, involving microhomologies, is also possible. Intermediates were discovered that suggest the existence of an unusual integration into the genome at the nick of one end of the fragment and the other end of the fragment hanging freely into the interchromosomal space. A direct assessment of the possibility of integrating TAMRA-labeled fragments of fragmented human DNA and E. coli DNA into the genome of recipient cells was carried out using a human bone marrow cell model. The results obtained indicate that specific signals of homologous DNA are distributed throughout the chromosome body (human bone marrow cell model). Signals from nonhomologous E. coli DNA are predominantly concentrated in the centromeric regions of chromosomes. The ratio of the number of obtained reads with integration elements and FISH signals suggested the existence of a strong interaction between extracellular fragments and chromosomal DNA. Experiments have been conducted showing that linear plasmid DNA, after internalization into hematopoietic stem cells, forms a monomer ring. Internalized into the intracellular space, extracellular plasmid DNA is isolated together with chromosomal DNA after stringent purification and fractionation procedures. This fact suggests the existence of a strong ring associate of plasmid DNA and chromosome DNA formed without the participation of a protein framework in the form of a looped chromosomal strand.

## Abstract (ru)

Для оценки возможности интеграции в реципиентный геном гемопоэтических стволовых клеток экстраклеточных фрагментов двуцепочечной ДНК был сконструирован сложно составленный субстрат, состоящий из целого М13F-AluI-M13R фрагмента и двух его производных, появляющихся после гидролиза рестриктазами EcoRI и HindIII: M13F-AluI-EcoRI и М13R-AluI-HindIII. В субстрате была представлена последовательность полилинкера плазмиды pBlueScript+, отсутствующая в геноме человека, которая обрамляла клонированный по сайту EcoRV AluI фрагмент человека. Клетки костного мозга человека были обработаны ДНК сконструированного сложно составленного субстрата, и с учетом времени репарации пангеномных одноцепочечных разрывов из клеток выросших колоний получены препараты метафазных пластинок. Проведенная FISH выявила специфические сигналы свечения. Одновременно ДНК, выделенная из колоний, полученных из клеток костного мозга, обработанных сложно составленным субстратом, была секвенирована. Проведено два раунда секвенирования: полногеномное и селективное после таргетной гибридизации на металлических бусах. Полученные результаты свидетельствуют, что гомологичный обмен между экстрахромосомальной и хромосомной ДНК возможен. Также возможна интеграция в геном по механизму однонитевого отжига, с участием микрогомологий. Обнаружены интермедиаты с одним концом фрагмента, интегрированным в геном по участку микрогомологии, и другим концом фрагмента, свободно свисающим в межхромосомное пространство. Проведена прямая оценка возможности интеграции TAMRA-меченых фрагментов двуцепочечной ДНК человека и E. coli в реципиентный геном на модели клеток костного мозга человека. Обнаружено, что специфические сигналы гомологичной ДНК распределены по телу хромосом (модель клеток костного мозга человека). Сигналы негомологичной ДНК E. coli преимущественно сконцентрированы в центромерных районах хромосом. Соотношение количества полученных ридов с элементами интеграции и сигналов FISH предполагало существование прочного взаимодействия экстраклеточных фрагментов и ДНК хромосом. В экспериментах показано, что линейная плазмидная ДНК после интернализации в гемопоэтические стволовые клетки формирует кольцо мономера. Интернализованная во внутриклеточное пространство экстраклеточная плазмидная ДНК выделяется совместно с ДНК хромосом после жестких процедур очистки и фракционирования. Этот факт предполагает существование прочного кольцевого ассоциата ДНК плазмиды и ДНК хромосом, сформированного без участия белкового каркаса в форме закольцованной хромосомной нити.

## Introduction

This article, the fourth in a series, further develops the concept that extracellular double-stranded DNA (dsDNA) fragments – linear DNA molecules of variable origin and size (200–2,000 bp or more) – are naturally delivered to hematopoietic stem cells (HSCs). The internalization process is facilitated by the general positive charge of HSCs, a characteristic determined by specific proteins within the HSC glycocalyx (Dolgova et al., 2012; Petrova et al., 2022; Ritter et al., 2022). The mobilization and internalization processes involve proteins containing heparin-binding domains or clusters of positively charged amino acids. The introduction of dsDNA into the cell has been shown to induce genome-wide single-stranded DNA breaks, which subsequently result in terminal hematopoietic stem cell differentiation (Ruzanova et al., 2024).

The process of terminal differentiation has been demonstrated to be associated with the reorganization of higherorder chromatin structure (Ruzanova et al., 2024). This process triggers the activation of a recombinogenic situation in the cell, with its enzymatic machinery determining the appearance and repair of pangenomic single-chain breaks and leading the cell to the path of development of a specific hematopoietic sprout (Jacobson et al., 1975; Farzaneh et al., 1982; Johnstone, Williams, 1982).

The recombinogenic situation in the cell is defined as the state in which the mechanisms of chromatin DNA metabolic transformations are activated. These processes include the following: activation of supervising kinases, modulation of the cell cycle, induction of replicative stress, and activation of the repair and recombination mechanisms. The structure of the latter includes the enzymatic machinery of the mechanism, as well as DNA substrates that form metabolic intermediates of the DNA core. Cumulatively, in the event of pangenomic single-strand breaks, this process is instrumental in ensuring the restoration of continuity and the integrity of the primary DNA sequence of chromosomes (Likhacheva et al., 2008; Ruzanova et al., 2024).

The introduction of DNA fragments into the cell has been shown to initiate nick formation (Ruzanova et al., 2024), leading to a recombinogenic situation and triggering extensive cellular alterations. The molecular mechanisms that initiate this process, as well as the causal relationships among the initiating factors, remain to be elucidated.

The research on the intracellular behavior of extrachromosomal, non-viral DNA commenced in the 1980s (Smith, Berg, 1984). Nowadays, this subject is not a prevailing area of research. The existing body of research provides an incomplete account of intracellular events triggered by dsDNA fragment entry and the resulting activation of cellular surveillance mechanisms.

Cell cycle progression control systems were reported to be activated in response to the appearance of DNA in the form of synthetic oligonucleotides, SV40 DNA, and apoptotic cells in the nuclear space (Yakubov et al., 1989; Holmgren et al., 1999). Additionally, mechanisms underlying the internalization of plasmid DNA through artificial means were described (Smith, Berg, 1984; Lin J. et al., 1985; Thomas et al., 1986). Several studies demonstrated cellular checkpoint responses to be activated upon the internalization of synthetic (dA/dT)70 oligonucleotides within the nucleus. The induction of cell cycle control systems in the absence of any genomic DNA damage or aberrant replication suggests that the structures of synthetic paired oligonucleotides may mimic intermediates arising from aberrant replication or genomic DNA damage, such as single-stranded DNA, blunt double-stranded ends, single-stranded site-duplex junctions, and cruciform shapes. The process of oligonucleotide pairing is assumed to lead to the creation of DNA molecule ends from paired fragments. These fragments function as inducers of ATM/ATR-dependent checkpoint cellular response through a mechanism that relies on regulators of hierarchical kinase activity such as RPA, RAD1, TopBP1, and claspin (Yoo et al., 2004, 2006; MacDougall et al., 2007; Zou, 2007). Studies examining molecular processes involved in DNA uptake indicate that single-stranded DNA fragments do not trigger cellular regulatory mechanisms (Kumagai, Dunphy, 2000). The analysis of the minimum number of linear molecules capable of inducing a checkpoint response demonstrated that more than 30 molecules containing ssDNA/duplex structures activated a sufficient checkpoint response required to determine the level of Chk1 phosphorylation (MacDougall et al., 2007). However, with the presence of fewer than 30 linear DNA fragments located in the extrachromosomal space of the nucleus, the cell stress monitoring system remains quiescent. Consequently, this quantity of linear DNA molecules may enter the nucleus without activating the hierarchical kinase system, suggesting that the transient presence of extracellular fragments circulating in the plasma is the norm.

Extracellular linear dsDNA fragments within the cell have been observed to undergo a process of cross-linking, resulting in the formation of concatemeric and ring structures with a size range of up to 10 kbp (Perucho et al., 1980; Lin J. et al., 1985; Lin Y., Waldman, 2001; Rogachev et al., 2006; Potter et al., 2017, 2024). Furthermore, it has been observed that linear plasmid DNA fragments located in HSCs are processed to a depth of up to 1 kbp (Dolgova et al., 2013). Consequently, the collective evidence suggests that the presence of extracellular dsDNA fragments within the cell initiates cellular repair and recombination pathways. This assertion is supported by experimental evidence (Pierandrei et al., 2016). RT2 Profiler PCR Array platform (SABioscience, Qiagen) was utilized to analyze the expression of 84 genes during the transfection of extracellular dsDNA fragments into the cell. The fragments of dsDNA that were delivered to the cell were found to activate genes of various cellular systems, including those involved in DNA repair and homologous recombination, as well as genes regulating the cell cycle and epigenetic modifications. In summary, the principal molecular systems responsible for regulating cellular DNA metabolism are subjected to a stressed condition.

Nicks, as well as the double-stranded ends of extracellular fragments delivered to the nucleus, were found to serve as triggers for repair and recombinogenesis (Vriend, Krawczyk, 2017; Maizels, Davis, 2018). While sharing some overlapping signaling pathways, the mechanisms and factors triggered by these two structures differ (Ruzanova et al., 2024). Consequently, the presence of two DNA metabolites (nicks in the DNA of chromosomes and double-stranded ends of extracellular fragments delivered to the nucleus) in the cell triggers unidirectional repair and recombination processes. Apparently, the factors of both processes are likely to be involved in certain molecular relationships that have yet to be disclosed. A comprehensive analysis is required to assess the viability of such a scenario within the framework of the concept under consideration.

The introduction of fragments into cells using a variety of transfection techniques constitutes a standard procedure. The most common technique is the knockout method. Three independent researchers, Mario R. Capecchi, Oliver Smithies, and Sir Martin J. Evans, won the Nobel Prize in 2007 for this approach. This method relies on the principle that homologous dsDNA fragments within the nucleus will recombine, resulting in a knockout mutation. Consequently, the process of the natural exchange of genetic information between extrachromosomal DNA and chromatin DNA can be objectively confirmed to exist. This exchange represents an intrinsic property of the metabolic activity of the cell, associated with the interaction of chromatin DNA and extrachromosomal DNA (Perucho, Wigler, 1981; Kucherlapati et al., 1984; Smithies et al., 1985; Murnane et al., 1990; Hastings et al., 1993; Leung et al., 1997; Li et al., 2001; Lin Y., Waldman, 2001).

Following their entry into the nucleus, the dsDNA fragments then engage in the processes they trigger. The introduction of recombinogenic nicks and double-stranded ends into the nucleus will inevitably initiate recombination processes, which we hypothesize to result in the emergence of new genetic information within the cell stored in the internalized dsDNA fragments of initially extracellular localization.

The present part of the study evaluates the possibility for extracellular DNA fragments to be integrated into the genome of HSCs and for ring-like associates to be formed between fragment DNA and chromosome DNA.

## Materials and methods

**Human bone marrow cells. **In this study, we used cells from cryopreserved bone marrow separates of Hodgkin’s lymphoma patients provided by the cryobank of the Research Institute of Fundamental and Clinical Immunology (RIFCI). The Clinic of Immunopathology of RIFCI (the Department of Hematology equipped with a bone marrow transplantation unit) provides the treatment of patients with hemoblastosis using high-dose chemotherapy and transplantation of autologous or allogeneic peripheral HSCs. When harvesting peripheral stem cells, along with the main apheresis product (which is transplanted to the patient), two or three samples (satellite tubes) of separated cells are prepared for quality control of the apheresis product and scientific research. Such samples were used in the present work along with the main sample. Comprehensive documentation, including patient-signed informed consent and approved protocols for bone marrow research and treatment, is provided for each bone marrow separate sample and satellite samples, adhering to established regulatory guidelines. Subsequent to treatment and use of the primary product, satellite samples undergo disposal conforming to Sanitary Rules and Regulations or are allocated for scientific applications. Documentation pertaining to each bone marrow sample is archived in the RIFCI cryobank and is readily available upon request.

***AluI *double-stranded DNA probe. **The amplification of the human AluI repeat (M13F-AluI-M13R fragment) was performed by means of PCR. The matrix was the repeat DNA cloned in plasmid pUC19 incorporating the start and the end of the tandemly repeated AluJ and AluY sequences (NCBI: AC002400.1, 53494–53767) (Dolgova et al., 2012). The amplification was performed using either standard M13 primers (M13 for: 5ʹ GTAAAACGACGGCCAGT 3ʹ, M13 rev: 5ʹ CAGGAAACAGCTATGAC 3ʹ) or AluI-specific primers (Dolgova et al., 2012). The PCR fragment was resuspended in 0.1 V NaAc 3 M pH 5.2 and 1 V isopropanol for 10 min at –20 °C, followed by centrifugation. The precipitate was washed in 70 % ethanol and dissolved in sterile water.

**DNA preparations. **The hDNAgr preparation (a human genome DNA reconstructor) is a human double-stranded DNA in the form of 200–2,000 bp fragments. The hDNAgr preparation was isolated from the placentas of healthy women, fragmented by ultrasonic disintegration to the size of 200–2,000 bp, deproteinized by proteinase K treatment, and isolated by phenol/chloroform extraction.

The human DNA was isolated from the placentas of healthy women using a similar method without fragmentation

The E. coli DNA was isolated in a standard manner using lysozyme, ultrasonic cell disruption, proteinase K deproteinization, and phenol/chloroform extraction

**Design of a probe for bone marrow cell processing.** For analyzing potential nonhomologous integration and/or homologous exchange, a probe was constructed consisting of an AluI fragment embedded in the pBlueScript+ (pBS) polylinker by the EcoRV site and restricted to sequences extending to and including the M13 primer landing sites. The internalization experiments involved using a mixture of the whole M13F-AluI-M13R fragment and two of its derivatives, generated through EcoRI and HindIII restriction enzyme hydrolysis: М13F-AluI-EcoRI and M13R-AluI-HindIII.

For analyzing possible direct integration, fragmented human and E. coli genomic DNA was tagged with a nucleotide containing the TAMRA fluorochrome using a random sequence primer (stat primer) in an amplification reaction with Taq polymerase (Medigen LLC).

The internalization analysis of the TAMRA-tagged M13F-AluI-M13R PCR fragment into Ehrlich carcinoma cells was performed according to the procedure described in (Ruzanova et al., 2022).

**FISH **was performed according to a standard procedure using TAMRA-labeled probes using the method described in Supplementary Material 11. The examination of FISH results was performed with an AXIOSKOP 2 Plus microscope (Zeiss, Germany). Microimage recording and processing were performed using a CCD camera (CoolCube 1, METASystems, Germany), Set 49 filter set (G 365, FT 395, BP 445/50) (Zeiss, Germany), ET-orange #1 (ET 546/22 × ET 590/33) (CHROMA, USA), and ISIS5 software (METASystems GmbH, Germany). The number of metaphases analyzed ranged from 14 to 30 metaphase plate images

Supplementary Materials are available in the online version of the paper: https://vavilov.elpub.ru/jour/manager/files/Suppl_Oshikh_Engl_30_2.pdf


**Bone marrow cell cultivation within a methylcellulose medium.** Brain tissue samples were procured via percutaneous aspiration from the RIFCI cryobank. Standard thawing methods preserve up to 90 % viability of bone marrow cells, confirmed by FACS analysis to include CD34+ cells, eliminating the need for additional verification.

Human bone marrow cells contained in the aspirate were washed with RPMI medium and introduced into the experiment. Control cells and probe-treated cell samples were precipitated for 10 min at 400g and resuspended in IMDM + 2 % FBS medium. Myeloid progenitor quantification and analysis were performed using MethoCult H4034 methylcellulose medium (Stem Cell Technologies) for both treated and control cell samples. Cell culturing took 10 to 15 days, on average, depending on the purpose of the experiment. Cells were analyzed and isolated from methylcellulose medium after cultivation according to the manufacturer’s instructions.

**Targeted sequencing.** Sequencing was performed using DNA isolated from cells treated with the compound probe: М13F-AluI-M13R, М13F-AluI-EcoRI, and M13RAluI- HindIII. Library enrichment was performed by hybridization with biotinylated polylinker DNA of pBS plasmid immobilized on Dynabeads® Streptavidin magnetic particles (Life Technologies, USA) according to the method proposed by T. Maricic et al. (2010). The Illumina MiSeq platform (TruSeq® DNA Sample Preparation Kit (Illumina)) was used for sequencing using a MiSeq Reagent Kit v2 (300 cycles).

**Whole-genome sequencing. **Sequencing was performed using DNA isolated from cells treated with the compound probe: М13F-AluI-M13R, М13F-AluI-EcoRI, and M13R-AluI-HindIII. A NovaSeq 6000 instrument (Illumina) with a read length of 2 × 150 bp was used. The Q30 value accounted for at least 90 %. TruSeq Nano DNA Library Prep kit (Illumina) was used for library preparation. Ultrasonic DNA fragmentation was performed on an ME220 Focused-ultrasonicator (Covaris). Capillary electrophoresis, performed using a TapeStation 4200 instrument (Agilent), was utilized for the assessment of DNA library quality. The Ugene and Blastn platforms were used to analyze the sequences obtained. Raw sequence data, stored in FASTQ format, are accessible upon request.

**Ring formation by extracellular dsDNA fragments internalized by HSCs and their closest progeny.** A detailed schematic of the experimental design is described in the “Experimental proof of ring formation by long dsDNA fragments internalized in HSCs and their looping of the chromosome DNA strand” subsection. All the experimental procedures were performed according to the protocols described in practical manuals (Maniatis et al., 1984; Glover, 1988)

The bone marrow cells were treated with DNA preparation, washed of DNA, and incubated for 2 hours in a CO2 incubator in a complete RPMI nutrient medium. Then, the cells were harvested and washed with physiological saline. The cytoplasmic membrane was solubilized with 0.2 % Triton X-100, layered on a 10 % sucrose gradient, and centrifuged at 500 g for 20 min. Saline-washed nuclei underwent lysis with 1 % SDS and 50 mM EDTA. Deproteinization was accomplished by treating with 200 μg/ml proteinase K at 58 °C for two hours, followed by phenolchloroform extraction. The resulting lysate was applied to a 10–30 % NaCl stepwise (5 %) gradient and centrifuged at ~300,000g (L-8 centrifuge, SW 50.1 rotor, 47,000 rpm) for 2 hours at room temperature. The scheme displays DNA molecular weight markers located within the designated percentage gradient zones near the last tube on the right (Glover, 1988; Dolgova et al., 2013). The 7th region of the gradient contains DNA with a size greater than 40 kbp, forming a precipitate at the bottom of the test tube. Precipitation of the DNA supernatant was achieved through the addition of an equal volume of isopropanol, prepared from a 0.3 M sodium acetate solution (pH 4.8), and incubation at –20 °C overnight. Following centrifugation and saline washing, fractions 1–6 were dissolved in 36 μl of H2O. Following a 70 % ethanol wash, the DNA precipitate from fraction 7 was subsequently dissolved in 36 μl of H2O. 12 μl of material (1/3 of the total volume) was spread on an agarose gel.

Sample 7 (sediment at the bottom of the gradient) was analyzed, with a Qubit DNA concentration of 1 ng/μL. The total yield was 36 ng. Gradient analysis was performed using one-third (12 ng) of the sample. Quantification of pEGFP-N1 plasmid DNA in the kanamycin resistance gene region was accomplished via Real-Time PCR using another one-third of the sample (12 ng). The remaining third of the sample (12 ng) underwent a transformation into electrocompetent DH-5 cells, yielding a titer of 107–108.

The transformation of 12 ng of fraction 7 produced a count exceeding 50 large colonies and approximately 5,000 small colonies. All large colonies used for DNA isolation (8 colonies) contained the pEGFP-N1 monomer. Small colonies were not found to contain plasmid DNA. The DNA extracted from large-scale bacterial colonies was subjected to enzymatic hydrolysis using the restriction enzymes HindIII, EcoRI, and HaeIII.

## Results

**Detection of synthetic DNA substrate by FISH on metaphase plates obtained from colony cells**

**Fig. 1.**
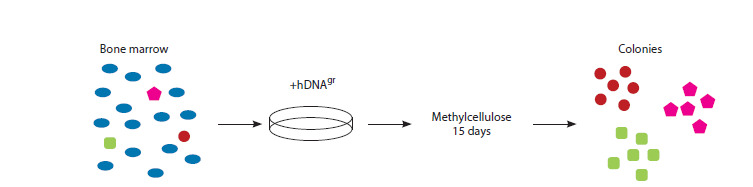
Experimental design overview

Colony formation, as described above, is a consequence of a single bone marrow hematopoietic stem cell undergoing division. Therefore, cells within a given colony exhibit identical genetic profiles, mirroring the genotype of the progenitor CD34+ HSCs (Fig. 1). Should the original HSC acquire genomic changes due to inducer treatment, these alterations will be amplified in the resulting colony to a detectable level, unlike the inherent difficulty of such detection within individual CD34+ bone marrow HSCs (property of LLC “ES LAB DIAGNOSTIC”).

The experimental protocol necessitated the creation of an artificial substrate for hematopoietic stem cell processing and a fluorescent probe to identify the integration of artificial substrate components. The options selected are detailed below. The amplified polylinker of the pBS plasmid was taken as foreign DNA, which is absent in the human genome. This DNA fragment, labeled with the TAMRA fluorophore, served as a hybridization probe (pBSM13-TAMRA+). Simultaneously, we used a complex substrate including an AluI repeat cloned at the EcoRV site, framed by the pBS polylinker, and ецщ fragments containing a portion of the pBS polylinker with an adjacent AluI repeat and bounded by EcoRI or HindIII restriction sites (M13F-AluI-M13R, M13F-AluI-EcoRI, and M13R-AluIHindIII). The internalization of TAMRA was assessed via spectrophotometry, electrophoresis, and visualization in an Ehrlich carcinoma ascites cell model (a standard procedure for assessing the fact of internalization). This approach to assessing DNA labeling in materials is a routine laboratory technique. Supplementary Material 1 provides a summary of the preliminary findings.

**Fig. 2.**
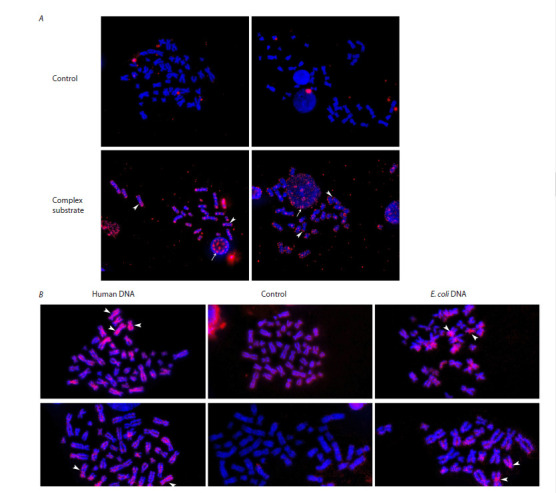
Analysis of the presence of specific FISH signals after treatment of bone marrow cells with the selected probe and specific signals detected after direct interaction of fluorochrome-labeled dsDNA probes and bone marrow cells on metaphase chromosomes. A – FISH with the pBSM13-TAMRA+ probe of metaphase chromosomes obtained after colchicine treatment of colonic cells grown from HSCs without treatment (Control) and treated with a complex substrate (M13F-AluI-M13R, M13F-AluI-EcoRI, and M13R-AluI-HindIII) as part of bone marrow cells. Arrows indicate multiple hybridization sites. B – distribution of TAMRA-tagged material on metaphase chromosomes derived from human colony cells after treatment of these cells with TAMRA-tagged human and E. coli DNA. The arrows indicate multiple localization sites of the tag in the sample treated with human DNA and centromeric regions in the sample treated with E. coli DNA.

The resultant construct was used to treat human bone marrow cells. On day 15 of methylcellulose cultivation, the colonies were harvested, washed free of the medium, and treated with colchicine. Then, metaphase preparations were prepared. Next, FISH with TAMRA-labeled probe pBSM13 (pBSM13-TAMRA+) was performed. Figure 2A presents the hybridization results. The preparations were found to exhibit multiple signals. The results obtained indicated a possible integration of the artificial probe into the recipient genome. Also, it was possible for extracellular DNA to be firmly associated with the chromosome body. When analyzing images of metaphase plates (up to 40), we did not focus on mapping the TAMRA signals. Therefore, we were not to achieve a complete representation of the karyotype in the images. The primary objective of the experiments was to ascertain the specificity of hybridization signals, which was confirmed through analysis of the resulting images.

Part of the cell samples used to obtain the metaphase plates under analysis were also used for targeting and full genomic sequencing (total DNA isolated from cells).

Furthermore, experiments employing fluorochromelabeled DNA probes were conducted to directly ascertain the presence of labeled material in (in association with) chromosomes. Human bone marrow cells, treated with TAMRA-labeled human and E. coli DNA, were cultured in methylcellulose. At 15 days, the cells were washed and treated with colchicine, followed by the metaphase plate analysis (Fig. 2B). The presence of specific signals was observed on a number of metaphase plates. In the case of human DNA, the labeled material was found to be detected at multiple sites on the chromosome body. In the case of bacterial DNA, the specific label proved to be concentrated to a greater extent in centromeric regions.

All the experiments with metaphase plates used metaphase preparations obtained from colony cells grown from a sample of bone marrow cells untreated with DNA substrate as control samples.

**Proof of recombination interaction of the artificial probe with the HSC genome using modern sequencing technologies**

In order to prove the recombination interaction of the М13F-AluI-M13R, М13F-AluI-EcoRI, and M13R-AluIHindIII DNA substrates with the genome of human HSCs, we employed the same cell material as used for metaphase preparation and FISH. We constructed a library and performed two types of sequencing: (1) whole-genome sequencing and (2) sequencing involving increasing the number of target fragments by enriching the library by hybridization with biotinylated polylinker DNA of the pBS plasmid included in the DNA substrate used, immobilized on Dynabeads® Streptavidin magnetic particles (Life Technologies, USA) according to the method of (Maricic et al., 2010). The results obtained were summarized in one section.

**Fig. 3.**
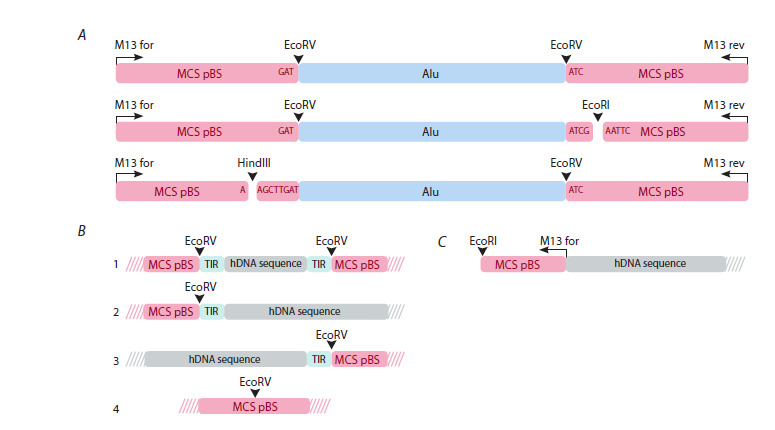
Schematic representation of the complex substrate used and several of its intermediates resulting from induced recombination processes. A – schematic representation of the substrate: the M13F-AluI-M13R, M13F-AluI-EcoRI, and M13R-AluI-HindIII fragments used for cell treatment. The original fragment and the same fragment hydrolyzed by EcoRI and HindIII restriction enzymes; B, C – schematic representation of sequencing-derived sequences

Figure 3A schematically depicts the substrates used for cell treatment and those present in the mixture. To sum up, the following derivatives of the original substrate were present in the mixture: M13F-AluI-M13R, M13F-AluI-EcoRI, EcoRI-M13R, M13R-AluI-HindIII, and HindIII-M13F. The sequences of nucleotides in the substrates are listed in Supplementary Material 2. Both forward and reverse orientations of the reads were analyzed for each chain.

*Nucleotide sequence analysis of the selected clones*

We identified the reads with distinctly cut-off ends terminating at the HindIII site and the primer sequences for M13 for. This indicates that the fragments from the original substrate are present in the cell in a “free state”. It should be noted that these are short fragments containing only part of the polylinker and the primer sequence for M13 for. The observed fragments may have survived 15 days of cell cultivation, localized either to a single quiescent HSC or dispersed amongst its progeny. Their size (<100 bp) may have shielded them from enzymatic processing, e. g., ring formation, concatemerization, or nuclease degradation (Supplementary Material 2).

A total of 56 sequences were found to contain a polylinker region from the pBS plasmid extending past the EcoRV site. Most of them were found to contain sequences flanked by the same inverted repeats (TIR) adjacent to both halves of the AluI cloning site of the EcoRV fragment (Fig. 3B1). Four reads had the AluI repeat replaced by an 84-nucleotide DNA sequence bounded on both sides by 10-bp terminal inverted repeats (TIRs) adjacent to the halves of the EcoRV cloning site (Fig. 3B2). The 21st read had the sequence terminating towards the left half of the conserved AluI EcoRV cloning site, failing to reach the opposite half of the EcoRV site, and bounded by the same inverted repeat (TIR) adjacent to the conserved left EcoRV site (Fig. 3B3). This 21st read is represented by three different sequences, one being 37 bp and the other two being approximately 100 bp in length and containing small regions (5–7 bp) of homology with the AluI repeat from the substrate.

Eleven reads include an intact full-length EcoRV site with no insertion, indicating the recombination of the AluI sequence cloned at the indicated site and the recovery of the original EcoRV (Fig. 3B4).

Fifteen reads match the original substrate containing part of the AluI repeatTwo reads were found with long (54 and 57 bp) regions of genomic DNA joined to the end of the M13 for sequence. These comprise a complete M13 for primer and a polylinker site terminating at a residual HindIII restriction site (Fig. 3C).

Furthermore, we identified reads exhibiting internal recombination at the AluI-EcoRV cloning site, those with chaotically shuffled polylinker sites, and one featuring DNA segments of non-substrate origin

*Experimental proof of ring formation by long dsDNA fragments internalized in HSCs and their looping of the chromosome DNA strand*

The apparent discrepancies in the number of fluorescent signals in FISH hybridization experiments, the findings of a direct analysis of the integration of extrachromosomal fragments into the genome, and the results of whole-genome sequencing suggested a potential mechanism for the formation of stable associates between DNA fragments localized in the nucleus and DNA strands of the chromosome. Given the lack of detectable large-scale single nonhomologous integration, a different mechanism is proposed for the formation of stable complexes. One type of associates that does not require protein anchoring is the DNA loop formed on the chromosome strand. This loop could have been formed by fragments of extracellular dsDNA delivered into the nuclear space. Experiments, largely replicating the methodology of a prior study (Dolgova et al., 2013), were performed to assess the assumption concerned.

**Fig. 4.**
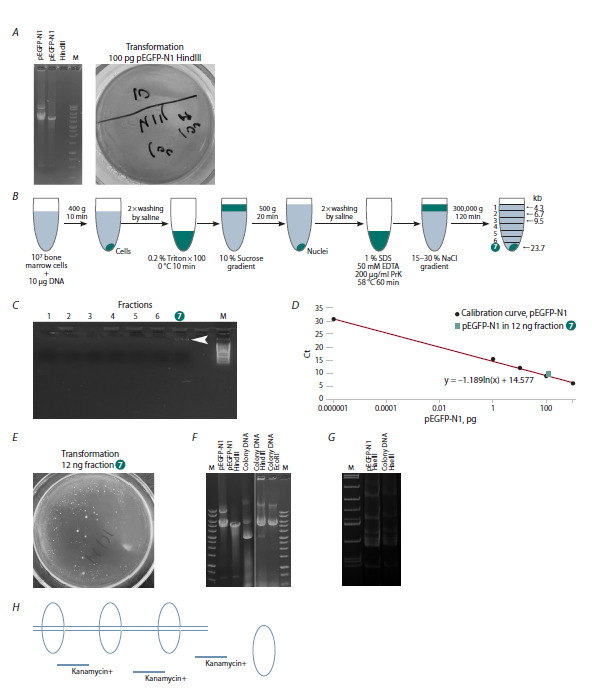
Experimental evidence of ring formation by long dsDNA fragments internalized in HSCs and their looping of the chromosome DNA strand. A – substrate of plasmid pEGFP-N1 used for processing of HSCs. Electrophoresis in agarose gel of native pEGFP-N1 plasmid hydrolyzed by HindIII. Transformation of pEGFP-N1 plasmid hydrolyzed by HindIII into E. coli, strain DH-5; B – the overall design of the experiment; C– electrophoresis of samples in 0.7 % agarose; D – analysis of the amount of pEGFP-N1 plasmid DNA in the kanamycin resistance gene region; E – transfection of 12 ng of fraction 7 resulted in more than 50 large colonies and about 5,000 small colonies; F, G – agarose and acrylamide gels with different variants of hydrolyzed and non-hydrolyzed plasmid DNA: the original pEGFP-N1 and one of the transformants (large colony). F – DNA isolated from large colonies was hydrolyzed by HindIII and EcoRI. Restriction analysis indicates intact HindIII and EcoRI polylinker sites. G – restriction analysis indicates an intact HaeIII monomer pattern and, thus, no intraplasmid recombination occurs. H – a putative schematic representation of the looped form of the plasmid formed around the chromosome after it enters the cell.

The experiments involved analyzing events and changes at the molecule level occurring with the substrate, plasmid pEGFP-N1, used for prolonged processing of HSCs (Fig. 4А). The analysis of the native plasmid sample revealed the presence of the dimeric form and the absence of the monomeric form. The transformation of 100 pg of linearized pEGFP-N1 (the amount equivalent to that obtained from the experimental sample DNA isolated from the bottom of the salt gradient, see below) into electrocompetent DH-5 cells yielded no transformants, thus confirming the absence of the ring form of the plasmid

Figure 4B presents a schematic overview of the experimental design. A detailed step-by-step description is given in the “Materials and methods” section, specifically in the “Ring formation by extracellular dsDNA fragments internalized by HSCs and their closest progeny” subsection.

An examination of fractions from high-speed saltgradient centrifugation by electrophoresis revealed the presence of DNA, as detected by ethidium bromide fluorescence, only in the final fraction (fraction 7, sediment) (Fig. 4C).

A quantitative analysis of the pEGFP-N1 plasmid DNA within the kanamycin resistance gene locus revealed that fraction 7 (12 ng) contained approximately 100 pg of homologous DNA (Fig. 4D).

The transformation of 12 ng of fraction 7 yielded over 50 macroscopic colonies and approximately 5,000 microscopic colonies (Fig. 4E). The macroscopic colonies were cultivated and inoculated in a liquid medium supplemented with 50 μg/mL kanamycin. The microscopic colonies exhibited growth in the liquid medium but did not develop further. No plasmid DNA in the form of monomer or dimer was found in the microscopic colonies. All the macroscopic colonies from which DNA was isolated (eight colonies) contained the pEGFP-N1 monomer. DNA isolated from the macroscopic colonies was treated with HindIII, EcoRI (Fig. 4F), and HaeIII (Fig. 4G) restriction enzymes.

Taken together, the results obtained in this series of experiments indicate the following:

• Bone marrow cells were treated with plasmid DNA linearized by the HindIII site. The presence of the original plasmid as a dimer and its monomer form in the resulting transformants suggests the recovery of the ring form linearized by HindIII plasmid within the bone marrow cells (BMCs). • Restriction analysis with HaeIII, HindIII and EcoRI confirms the absence of intraplasmid recombination (Fig. 4 F, G). • Stringent fractionation of plasmid and chromosomal DNA via NaCl gradient ultracentrifugation, following exhaustive proteinase K digestion and phenol-chloroform extraction, reveals a stable plasmid-chromosome DNA complex

The most likely scenario involves genomic DNA looping facilitated by the plasmid ring, which is formed during the linear pEGFP-N1 cyclization upon cellular entry (Fig. 4H).

The disparity between the pEGFP-N1 kanamycin resistance gene copy number, the number of transformants obtained, and the DNA yield, as determined through comparison, suggests the following explanation. Following transformation, multiple degraded plasmids, all possessing a kanamycin resistance gene, may have produced a single kanamycin resistance protein, manifested as small colonies. It is these fragments that were evaluated by quantitative real-time PCR. Analysis of large transformants indicates fewer than one plasmid DNA molecule per cell with DNA capture capability. Viable transformants may have resulted from plasmid rings released during chromosomal DNA isolation. The remaining portion, associated with chromosomal DNA, failed to yield transformants, thereby compromising the accuracy of quantification.

Our findings corroborate those presented by E.V. Dolgova et al. (2013), confirming the ring closure of internalized dsDNA fragments within HSCs in both experimental contexts. At the moment of closure, some rings seem to stochastically encompass the DNA strand of the chromosome, forming a stable complex.

## Discussion

**Fig. 5.**
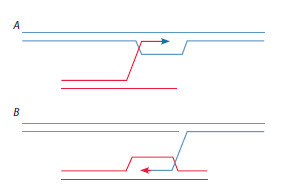
Schematic representation of recombination processes (single-strand annealing, gene conversion, microhomology pairing) occurring in the cell involving genomic DNA and dsDNA fragments delivered to the cell. A – recombination due to the open double-stranded ends of the delivered fragments; B – recombination due to single-strand breaks in genomic DNA.

The experimental system demonstrated recombination activity between chromatin DNA and double-stranded extrachromosomal DNA fragments, which functioned as interacting partners in this process. A number of publications characterize experimentally verified and theoretically predicted intermediates resulting from the recombination of chromosomal DNA, single-stranded chromosomal DNA breaks, and extrachromosomal double-stranded DNA fragments (Hastings et al., 1993; Leung et al., 1997; Cromie et al., 2001; Li et al., 2001). Figure 5 offers schematic illustrations of the aforementioned intermediates. The relationship may be categorized into two variants. The first scenario presents an active recombinogenic condition that generates an enzymatic mechanism for recombination. This can occur at any genomic site through the interaction of double-stranded fragment ends participating in a reciprocal exchange with homologous chromosome sequences (Fig. 5А). In the second scenario, it is the DNA strand termini exhibiting the nick that are recombinogenic (Fig. 5В).

This work explored the potential for incorporating extracellular DNA into the genome. Two methodologies were employed: FISH and whole-genome sequencing. In pursuit of this objective, a composite probe was developed, consisting of an AluI fragment as its core, framed by the left and right pBS polylinker sequences and a number of their derivatives. Hydrolysis with EcoRI and HindIII restriction enzymes yielded derivative fragments representing portions of the complete substrate. Figure 3A depicts a mixture containing five distinct elements. A hypothesis was formulated suggesting that the successful association of probe and chromosome DNA would result in the observation of specific signals on metaphase chromosomes through the application of FISH

The structured nuclear hybridization signals revealed through image analysis might demonstrate specific characteristics. The FISH signal indicated potential homologous and nonhomologous substrate integration into the genome. An alternative hypothesis regarding the origin of specific signaling was proposed. This phenomenon was associated with the integration of extrachromosomal elements into actively transcribed chromatin regions, resulting in the formation of stable chromatin complexes between extrachromosomal and chromosomal DNA, persisting until metaphase (Møller et al., 2018; Wu et al., 2019; Zhu et al., 2021; Pecorino et al., 2022). This issue is considered in the last part of the “Discussion” section.

Whole-genome and selective sequencing methodologies were implemented to test the stated hypotheses. These methods enabled the identification of two intermediate variants, suggesting recombination between extracellular dsDNA and nuclear chromosome DNA fragments

**Fig. 6.**
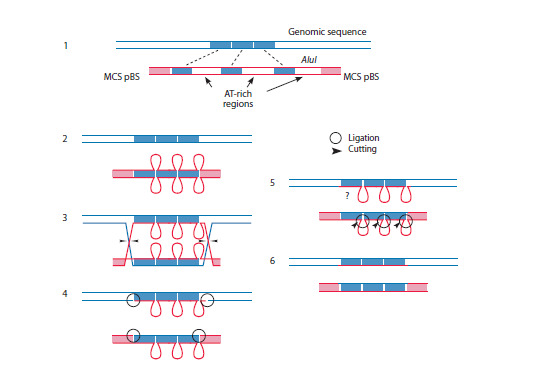
Schematic representation of possible events during the formation of one of the detected intermediates identified by analyzing the full-genome sequencing reads. 1 – genomic DNA and substrate delivered to the cell. Homologous regions are highlighted in blue; 2–4 – sequential steps of recombination events, with regard to AT looping of AT-rich nonhomologous sites; 5, 6 – predicted recombinant products, with sequencing data included

1. Over 20 genomic DNA sequences with discernible AluI fragment substitutions were detected using EcoRV cloning sites for identification. The genomic DNA segment extends significantly from the AluI cloning site of the EcoRV fragment on both its left and right flanks, or it is positioned between the two halves of the EcoRV cloning site. Without exception, this genomic segment is situated adjacent to the halved EcoRV cloning site, with the boundary defined by specific sequences constituting a 10 bp terminal inverted repeat (TIR). In some reads, this fragment exhibits homology with cloned AluI, while others contain small regions (5–7 bp) of homology with the AluI repeat of the substrate (Fig. 6).

The TIR is known to flank the sequence of the mobile genetic element hAT-5_RIr. hAT elements are a superfamily of DNA transposons that are moved around in the genome by the transposase enzyme. Their presence has been confirmed within the genomes of plants, fungi, and animals. In mammals and humans, however, these elements exhibit no activity. They are approximately 2.5–5 kbp in size and contain terminal inverted repeats (TIR). The analysis of the structures of the final intermediates reveals that the complete substitution of the AluI repeat of the original substrate for the DNA sequence bounded by terminal inverted repeats occurred exactly on the flanks of the AluI repeat, a mobile genetic element of the human genome (Hagan et al., 2003). One intermediate, in which substitution preserved both halves of the AluI-EcoRV cloning site, exhibits clear homology with the AluI substrate.

Central to the analysis is the observation that, in all instances, the centrally located AluI nucleotide sequence has been replaced solely by genomic sequences at the EcoRV cloning site. Recombination was limited to homologous sequences between the genome and the AluI substrate, with nonhomologous plasmid DNA excluded. The analysis revealed no reads in which plasmid DNA sequences were enclosed by genomic DNA sequences, thus suggesting nonhomologous insertion events. A logical interpretation of recombinational events in a read, showing complete preservation of substrate structure and complete replacement of the genomic sequence with the AluI DNA sequence, is depicted in Figure 6

**Fig. 7.**
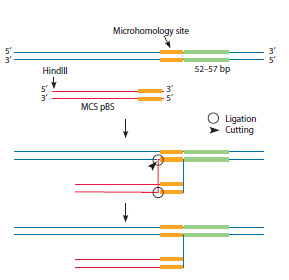
Schematic representation of possible events during the formation of intermediates detected when analyzing the results of full-genome sequencing, in which a section of genomic DNA is combined with a plasmid polylinker. It is suggested that recombination occurs at the site of a single-stranded genomic DNA break by a single-stranded annealing mechanism due to complementary microhomology pairing. The genomic DNA sequence is indicated in blue. The green color indicates the section of genomic DNA detected in the two derived reads. The yellow color indicates the microhomology section. The pBS polylinker (MCS pBS) is indicated in red.

2. Two reads were identified, with the right side of the hydrolysate (HindIII-primer M13 for) of the starting fragment being in the same continuous sequence as the human genome segment (54 and 57 bp), joined through the M13 for and genome microhomology site (Fig. 7). Moreover, the second part of both reads terminates with a cut-off strictly at the HindIII restriction site, which is the “terminus” of the substrate. It should be noted that the genomic DNA region of the two reads is 95 % homologous, implying that the integration may have occurred either in different parts of the genome or in the same part of the genome but in different cells where the acceptor sequences are not 100 % homologous.

A hypothesis is proposed to explain the discovered structure. The genomic sequence of the intermediate experienced a single-stranded break at its outset, or four letters before the start of the genomic sequence of the hybrid read. In this region, four nucleotides within the genomic sequence are complementary (homologous) to the four terminal nucleotides of the M13 forward primer. The fusion of extrachromosomal and genomic DNA within the polylinker matrix resulted from single-strand annealing, a process initiated by microhomology-based complementary pairing and D-loop formation (McVey, Lee, 2008; Hastings et al., 2009). The characteristic feature of both read sequences is their strict termination at the HindIII restriction site. The probability of the original DNA undergoing a precise, HindIII-clear double break during simultaneous ultrasonic processing of two fragments is rather low. This finding demonstrates that HindIII-terminated tails are extranuclear and persist freely in the nucleus for 15 days without genomic integration. Figure 7 illustrates potential intermediates from the aforementioned process.

So, intermediates were found that indicate the possibility of homologous exchange between extracellular DNA and chromatin DNA.

Additionally, unusual structures were identified that exhibited covalent attachment at one terminus to the recipient genome, while the opposing terminus was cleaved at the restriction site employed in substrate preparation (Fig. 3C). The data suggest that this part of the sequence is either free within the nucleus, lacking association with the second DNA strand, or is associated with a protein structure required for chromatin compaction into chromosomes and subsequent cell cycle events. We hypothesize that the observed association in these cases resulted from microhomology-driven interactions between genomic and M13 primer DNA at the single-strand break.

The identification of several intermediates is indicative of substrate DNA recombination. The identified reads exhibited multiple, chaotic permutations of both substrate and non-substrate sequences. These findings further support the occurrence of active recombination within the cell.

**Schematic representation of the sequence of events and intermediate variants occurring during the act of homologous recombination**

**An intermediate resulting from recombination of an internal AluI repeat cloned at the EcoRV site and framed by polylinker regions bounded by primers to M13 with genomic DNA.**

The genomic DNA sequence located after the first terminal inverted repeat (TIR) is homologous to segments of the AluI repeat that are separated by AT-rich regions (spacers) (Fig. 6). A possible explanation for the convergence of the AluI spacer-containing substrate sequence to a homologous genomic region involves the looping-out of AT-rich spacer sequences, followed by the linear convergence of AluI substrate segments exhibiting homology with the genomic DNA. In this scenario, it is possible that homologous recombination took place between the genomic sequence and the substrate used in the cellular treatment. This case involved the complete replacement of the AluI repeat element in the extrachromosomal DNA with a genomic DNA fragment. Such events can arise from reciprocal homologous recombination at either open chromatin sites or arbitrary genomic locations. This result indicates an active process of homologous precisional recombination between extracellular DNA and chromatin DNA.

**A part of the polylinker that terminates at the HindIII restriction site at one end and is joined at the other end to DNA of genomic origin by microhomology.**Geno- mic restructuring in differentiating cells is initiated via pangenomic single-strand breaks, which resolve within one week. The observed intermediates appear to correlate with the presence of single-stranded DNA breaks concurrent with the nuclear entry of extracellular DNA probe fragments (Fig. 7).

The situation allows for two possible interpretations. The integration event may have occurred during the initial committing phase, with intermediates emerging and existing prior to metaphase formation. The intermediary steps may have taken place concurrently with, or shortly prior to, DNA isolation on day 15 of cell culture. This variant involves the prolonged retention of short substrate fragments in the nucleus space. The retention of intermediate products throughout the duplication process is not clearly established. Considering the integration, it is probable that embedding occurred following chromosome doubling at those sites. The significance of such post-replicative disruptions to chromatin condensation and metaphase chromosome formation is seemingly negligible. The polymerase is unlikely to adopt such intermediate conformations during replication, which would require subsequent correction by specialized complexes.

The genomic DNA regions in both strains share 95 % homology, as previously stated. This indicates integration events at either different genomic loci or at the same locus in cells with non-identical acceptor sequences

**Fig.8.**
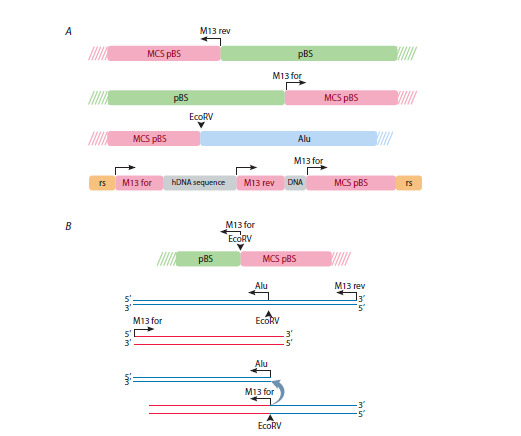
Schematic representation of sequences obtained by sequencing (A) and a possible variant of the reparative process (B).

**Reads with multiple chaotic permutations of substrate sequences and non-substrate sequences.**Figure 8 presents a schematic representation of sequences exhibiting multiple chaotic permutations generated through sequencing.

In the last series of experiments, the feasibility of integrating extracellular fragments into the genome by direct detection of the integrated fragment was evaluated. Two types of TAMRA-labeled DNA, homologous human DNA and E. coli DNA, were used. The main conclusion of these experiments is that, in the case of human DNA, specific signals are detected throughout the chromosomes. In the case of E. coli DNA, these signals were found to be more localized in centromeric heterochromatin. Assuming that integration into the genome has occurred, the mode of this integration will be different for homologous and heterologous DNA, namely homologous incorporation of the human substrate and nonhomologous integration of E. coli DNA. Centromeric heterochromatin is a bulk (conglomerate) of repeats corresponding by mass to 1/3 of the chromosome mass, creating its known three-dimensional architecture. Assuming integration into centromeric heterochromatin, the considerable size and specific organization of α-satellites suggest that the addition of DNA does not compromise the functional integrity of the centromeric locus. This observation may clarify the process by which heterologous DNA is integrated

**Discrepancies between the results of FISH, direct detection of the fluorescently labeled probe at metaphases, and the data of whole-genome sequencing**

An analysis was conducted on the results of FISH, i. e. direct detection of possible integration of a fluorescencelabeled probe into the genome, and data of full genomic sequencing in the experiments performed using a probe consisting of a mixture of M13F-AluI-M13R, M13F-AluIEcoRI, and M13R-AluI-HindIII fragments. This analysis revealed the apparent discrepancy between the number of hybridization signals, or simply specific fluorescent signals, and the number of reads demonstrating the actual covalent association of the substrate sequence and the chromosome sequence. The most apparent initial assumption attributed the result to methodological artifacts. However, analysis of different experimental variants, and in particular, the nuclear signal patterns, indicated atypical and specific signal characteristics. Therefore, supplementary experiments were undertaken in an attempt to clarify the underlying mechanism of metaphase signaling.

The findings have redirected our focus to a currently debated phenomenon: the presence of substantial ring-shaped extracellular DNA (Pecorino et al., 2022).

The study of naturally occurring extrachromosomal circular DNA (eccDNA), abundant in diverse tumor cell types, commenced over six decades ago with the identification of double minutes, circular extrachromosomal structures lacking centromeres and telomeres (Spriggs et al., 1962). The lack of centromeres leads to a random distribution of extracellular DNA during cell division, with the information encoded in them being transmitted in a non-Mendelian manner. These structures are formed from chromosomal material. Two structural categories are identified, the first characterized by single DNA sequences, and the second, by a chimeric structure composed of multiple chromosomal fragments. This observation indicates that chromosomal DNA fragments, detached through undetermined mechanisms, are randomly joined to form ring structures. Some of these DNA structures are up to 1 million bp in size and contain numerous genes and regulatory regions. Micro extrachromosomal circular DNAs of 100–2,000 bp in length are more prevalent in healthy cells. These DNAs contain ribosomal genes, DNAs of mobile elements, and telomeric ring DNAs. In tumor cells, these extrachromosomal structures can reintegrate into the genome, with “non-native” chromosomal regions with homogeneous staining containing amplified genes (Pecorino et al., 2022). Extrachromosomal circular DNA has been found to bind closely to the polymerase complex (RNAPII) and is thought to associate with actively transcribed chromosome domains with which it forms chromatin concentration foci (Wu et al., 2019; Zhu et al., 2021). This finding suggests that associations of extrachromosomal ring DNA and chromosome DNA are stable and can persist in various phases of the cell cycle.

Similar DNAs were reported to be present in human myocytes and leukocytes, indicating that these structures appear to be a common extrachromosomal object and represent a general biologic phenomenon (Møller et al., 2018).

Taking into account the above, it can be hypothesized that in our study, the probe containing the plasmid tag formed chromatin concentration foci with chromosomes without integrating into the chromosome DNA molecule. These associates persisted until metaphase and gave rise to multiple specific hybridization signals. Is there consistency between this assumption and the data obtained? Is such a form of intermediates possible?

The findings of whole-genome sequencing indicate that homologous probe integration is possible through either microhomology pairing or homologous recombination in the analyzed case of the AluI sequence. However, the number of reads-integrates obtained does not correspond to the hybridization pattern, with numerous specific signals (as initially assumed hybridization sites) detected at metaphases and, especially, concerning their patterning in interphase nuclei. Additionally, the results of direct integration analysis using fluorescently labeled homologous and heterologous probes, showing multiple specific signals (Fig. 2), require further clarificationThere is a lack of consistency between the quantified sequencing data and the observed hybridization pattern. Sequencing necessitates 40 ng of DNA, representing approximately 4,000 nuclei. Cumulatively, two repeats of the whole-genome reads revealed approximately 50 tagcarrying reads for each sequence. Thus, there is one substrate molecule per 80 cells. This is also inconsistent with the hybridization pattern, as multiple specific signals (hybridization signals) are detected at both metaphase and interphase nuclei (Fig. 2).

The facts and their analysis are more likely to suggest the formation of a large number of strong associates, which, together with the actual integration, give the overall FISH picture. The question remains as to why these multiple associations are not detected by full-genome sequencing. One suggestion is a technical problem.

The internalization of dsDNA fragments (nine telomeric repeats, 54 bp) into human and murine CD34 cells was experimentally confirmed, with results indicating that the dsDNA probe was successfully delivered to both CD34+ and CD34– cell populations (Ruzanova et al., 2024).

The Illumina platform full-genome sequencing technique involves fragmenting genomic DNA into 100–200 bp fragments. The quantitative likelihood of a 100–300 bp ring remaining intact following sonication is high. However, plasmid tag sequences (approximately 200 bp in this experiment) will not be detected, although they are fully present on the chromosomes. It is this assumption that we believe to account for the contradictory findings yielded by FISH, whole-genome sequencing, and direct detection of fluorescently labeled probe integration

A comparative analysis of the size of the ring formed by the oligonucleotide (about 350 bp) (Ruzanova et al., 2024), the diameter of it encompassing a linear molecule of the interphase chromosome, and the size of the polymerase complex suggests the possibility of the replicative enzymatic machinery moving freely through the ring during replication, with dsDNA strand thickness ~2 nm, one 10.5 bp dsDNA strand ~3.4 nm, 300 bp fragment ~100 nm, 300 bp dsDNA ring diameter ~32 nm, and polymerase complex ~300 kDa ~20 nm (https://www.dynamic-biosensors.com/ project/list-of-protein-hydrodynamic-diameters/). The ring structure remains intact until the metaphase stage, at which point it is detectable via FISH.

**Direct evidence for the long fragments of extracellular dsDNA (pEGFP-N1 linearized HindIII) forming a ring spanning the chromosome strand**

The objective contradictions and doubts raised prompted a necessary inquiry into the signaling processes occurring on metaphase chromosomes.

Our previous research (Dolgova et al., 2013) using the example of linearized plasmid pEGFP-N1 (4.7 kbp) demonstrated that once a recombinogenic situation is induced in HSCs (mouse model), triggered by double-strand breaks as a result of the cross-linking cytostatic cyclophosphamide (“death window” phenomenon), linear plasmid fragments internalized into these cells undergo metabolic changes. The concatamers arrange themselves into ring formations, with a maximum of four monomers per ring. A subset of fragments undergoes end hydrolysis to a depth of 1 kbp, maintaining this structure within the nucleus. Of key importance is the observation that plasmid ring forms are isolated along with chromosomal DNA at the bottom of a 15–30 % NaCl gradient (~300,000g) subsequent to exhaustive proteinase K digestion and phenolic extraction. Specifically, plasmid DNA interacts with chromosomal DNA within a rigidly fractionated environment, while lacking any proteinmediated structural linkage. Common sense suggests two possibilities: plasmid integration into the chromosome or plasmid circularization around the chromosome, both resulting in cosedimentation with genomic DNA in the salt gradient. The significance of this fact was not initially recognized during the results analysis.

The present study shows that, similar to the results of the cited work, in human HSCs, when extracellular dsDNA fragments enter them, a recombinogenic situation is induced, provoked by the nicks that these fragments initiated. A comparison of the results obtained in the second part of this study (Ruzanova et al., 2024) with the earlier findings (Dolgova et al., 2013) indicates that during the two recombinogenic situations with different origins (induced by double-strand breaks and nicks), after entering the cell, the extracellular fragments undergo similar changes at the first stage of metabolic transformations. Their concatamerization (cross-linking/ligation at the ends) possibly implies that all other transformations of the fragments in the nucleus are similar to those shown previously (Dolgova et al., 2013). In other words, the fragments are either concatamerized and/or form a ring, and in the process of cyclization in the stochastic mode, the DNA strand of chromosomes can be looped by the cyclized plasmid. We can thus conclude that all experiments examining metaphase chromosome fluorescence signals have implicitly identified these structures.

To validate the aforementioned hypothesis, a set of experiments was conducted, closely replicating those conducted earlier (Dolgova et al., 2013) (Fig. 4). With the exception of the cell model (murine vs human bone marrow), the key differentiating factor was the method of recombinogenic state induction. Previous studies utilized double-strand breaks (interchain cross-links), whereas the current investigation uses nicks. The results obtained were virtually indistinguishable. Upon entry into hematopoietic stem cells, the linearized plasmid undergoes cyclization via the cohesive HindIII ends, resulting in a ring structure. Cyclization involves plasmid-induced looping of the chromosomal DNA, which co-purifies with the chromosomal DNA fraction at the bottom of a 15–30 % NaCl gradient during density gradient centrifugation. This structure, a long-standing component of the nucleus, is likely detectable “as a constituent” of the metaphase chromosome (Fig. 4). This interpretation of events aligns with whole-genome sequencing data, revealing no evidence of nonhomologous large-scale integration resulting in multiple discernible signals on metaphase chromosomes

The processes governing the fate of these intermediate structures during metaphase chromosome packaging and division are yet to be elucidated. It can be hypothesized that ring structures undergo either physical fragmentation or enzymatic processing, resulting in linearization. Regardless, the stored genetic information may comprise an informational “reservoir” of currently undetermined function. The loosely arranged rings formed by extracellular dsDNA fragments may be analogs of rings that are involved in the mechanism of alternative telomere elongation.

## Conclusion

The occurrence of pangenomic single-strand breaks (nicks) consequently activates cellular repair and recombination processes, thereby inducing recombination between chromatin DNA and exogenous dsDNA. It is worth noting that no nonhomologous end joining (NHEJ) events were detected between extracellular/extrachromosomal substrate DNA and chromosomal DNA. These findings indicate the potential for homologous genome modification during this biological process while significantly reducing the likelihood of nonhomologous, random, and functionally detrimental integrations. Extracellular DNA fragments trapped in HSCs concatamerize, form rings, and establish long-lasting spatial associations with chromosomal DNA. It is possible for extrachromosomal rings to be the DNA matrix used by the cell in the mechanism of alternative telomere elongation. Finally, extracellular dsDNA fragments in the cell under the pressure of the recombination situation induced by both double-strand breaks and nicks undergo similar metabolic transformations, indicating the commonality and consistency of the two processes, which do not “conflict” and can complement one another.

## Conflict of interest

The authors declare no conflict of interest.
